# The role of autoantibodies in post-chikungunya viral arthritis disease severity

**DOI:** 10.1128/spectrum.02656-24

**Published:** 2025-03-05

**Authors:** Samantha Ansah-Dico, Ilana Heckler, Michelle Premazzi Papa, Alfonso Sucerquia Hernández, Jose Forero Mejía, Sarah Renee Tritsch, Evelyn Mendoza-Torres, Liliana Encinales, Andres Cadena Bonfanti, Abigale Marie Proctor, Jessica M. Wells, Daniela Díaz Hernández, Juan Manuel Pretelt Gazabon, Mónica Gómez Pulido, Sara Camila Castiblanco-Arroyave, Sammuel Joseph Simmens, Rebecca Lynch, Aileen Yu-hen Chang

**Affiliations:** 1 Department of Microbiology, Immunology and Tropical Medicine, George Washington University 8367 , Washington, District of Columbia, USA; 2 Department of Scientific Affairs, EUROIMMUN US 603221 , Mountain Lakes, New Jersey, USA; 3 Department of Medicine, George Washington University 571936 , Washington, D.C., USA; 4 Global Health, Milken Institute School of Public Health, George Washington University 50430 , Washington, D.C., USA; 5 Advanced Biomedicine Research Group, Faculty of Health, Exact and Natural Sciences, Universidad Libre de Colombia 469626 , Barranquilla, Atlantico, Colombia; 6 Department of Medicine, Allied Research Society, Barranquilla, Atlántico, Colombia; 7 Centro de Investigación, Clínica de la Costa SAS, Barranquilla, Atlántico, Colombia; 8 Department of Quality Operations, EUROIMMUN US 603221 , Mountain Lakes, New Jersey, USA; 9 Pediatrics Department, Faculty of Health, Exact and Natural Sciences, Universidad Libre de Colombia, Seccional Barranquilla, Barranquilla, Atlántico, Colombia; 10 Internal Medicine Department. Faculty of Health, Exact and Natural Sciences, Universidad Libre de Colombia, Seccional Barranquilla, Barranquilla, Atlántico, Colombia; 11 Microbiology Department. Faculty of Health, Exact and Natural Sciences, Universidad Libre de Colombia, Seccional Barranquilla, Barranquilla, Atlántico, Colombia; 12 Department of Biostatistics and Bioinformatics, Milken Institute School of Public Health, The George Washington University Milken Institute School of Public Health 50430 , Washington, D.C., USA; University of Miami, Miami, Florida, USA

**Keywords:** chikungunya virus, arthritis, arthritis disease severity, antibodies, autoantibodies

## Abstract

Post-chikungunya viral arthritis may persist for months to years after infection and is characterized by relapsing and remitting symptoms. This study investigates the relationship between autoantibodies and chikungunya arthritis severity, providing insights into arthritis pathogenesis. We assessed arthritis measures in a cohort of serologically confirmed chikungunya cases from Colombia between 2019 and 2021 (*n* = 144). We measured arthritis disease severity, flare intensity, pain, and disability, then plasma antibody levels of rheumatoid factor IgM, anti-cyclic citrullinated peptide (CCP), anti-citrullinated α-enolase peptide 1 (CEP-1), anti-nuclear antibody (ANA), anti-citrullinated vimentin (Sa), and immunoglobulins produced in response to chikungunya, Zika and Mayaro. Finally, we examined the correlation between the arthritis measures with the titers of antibodies hypothesized to play a potential role in arthritis pathogenesis. Cases were characterized by moderate disease severity (Disease Activity Score-28 mean, 3.66 ± 1.23) in current arthritis flare with moderate intensity (Flare Score, 25.42 ± 12.38), moderate pain (61.47 ± 27.23 on visual analog scale 0–100), and some disability (Health Assessment Questionnaire 0.77 ± 0.58). After Bonferroni adjustment, there were no statistically significant correlations between the levels of antibodies and arthritis measures. Weak correlations between rheumatoid factor IgM with arthritis severity and pain (*P* < 0.01) and anti-CEP1 with disability (*P* < 0.05) were observed when unadjusted for multiple comparisons. The data suggest that autoantibodies, such as RF, anti-CCP, and anti-CEP-1, do not correlate with post-chikungunya arthritis disease severity, thus unlikely to significantly contribute to pathogenesis. Exposure to other arboviral infections was not related to worse post-chikungunya arthritis. This suggests that other pathways for arthritis disease pathogenesis should be examined.

**IMPORTANCE:**

This cohort study describes the correlation between levels of autoantibodies, viral antibodies, and arthritis outcomes, suggesting that autoantibodies known to play an important role in other autoimmune diseases do not correlate with chikungunya arthritis relapse disease severity and are unlikely to contribute significantly to arthritis pathogenesis. This suggests that other pathways for arthritis disease pathogenesis should be examined to identify diagnostic and prognostic markers of alphaviral arthritis.

## INTRODUCTION

Chikungunya virus (CHIKV) is an alphavirus transmitted by mosquitoes that causes joint pain and arthritis, which can persist for months or even years (1, 2). The name “chikungunya” means “bent over in pain” in the Makonde language (3). CHIKV is present in 115 countries worldwide, with infection rates rising due to factors like unplanned urbanization and climate change, which impact mosquito and human population dynamics (3). Furthermore, CHIKV is one of several arthritogenic alphaviruses, including O'nyong-nyong virus (ONNV), Ross River virus (RRV), Barmah Forest virus (BFV), Mayaro virus (MAYV), Sindbis virus (SINV), and Semliki Forest virus (SFV) (4). Evidence-based therapies are lacking for all these alphaviral arthritides. Understanding the mechanisms behind chronic arthritis caused by CHIKV is a crucial step toward identifying therapeutic targets.

The arthritis following chikungunya infection is typically bilateral and symmetric, predominantly affecting the small joints of the hands, feet, wrists, and ankles (1, 2, 5). Post-chikungunya arthritis (CHIKA) is a debilitating condition that contributes to significant morbidity and loss of economic productivity (6, 7). The severity of CHIKA is comparable to that of rheumatoid arthritis (RA) (5), with notable similarities in gene expression profiles between the two conditions (8). In RA, autoantibodies such as rheumatoid factor (RF) play critical roles in diagnosis, prognosis, and pathogenesis (9–11). High RF titers are associated with more aggressive disease, characterized by erosive arthritis, extra-articular manifestations, and poorer clinical outcomes (9). RF can also promote immune complex formation at synovial sites, leading to complement activation and leukocyte infiltration (9, 12). However, the role of autoantibodies or antiviral antibodies in CHIKA pathogenesis remains unclear and warrants further investigation.

Prior studies have identified the prevalence of rheumatoid factor (RF) and anti-cyclic citrullinated peptide (anti-CCP) antibodies in CHIKA patients up to a prevalence of 57% and 29% in some cohorts meeting the criteria for rheumatoid arthritis after CHIKV infection (13), but it is unknown whether the presence or levels of these antibodies correlate with disease severity or key patient outcomes, like pain and disability. Additionally, multiple reports have shown the persistence of CHIKV IgM antibodies in CHIKA patients years after the initial infection (14–16), suggesting a potential link between persistent anti-CHIKV IgM antibodies and ongoing arthritic symptoms. However, it is unclear whether anti-CHIKV antibody levels are diagnostic of CHIKA, prognostic for severe arthritis, or play a role in its pathogenesis. The primary objective of this analysis is to rigorously evaluate a large cohort to determine the relationship between autoantibodies, antiviral antibodies, and CHIKV arthritis outcomes.

## MATERIALS AND METHODS

### Study design

Cases of clinically and serologically confirmed CHIKV infection aged 18 years and over from Magdalena and Atlántico Departments, Colombia, were enrolled between 2019 and 2021. An in-person history and physical examination were conducted to ascertain demographic characteristics, exposure history, and arthritis signs and symptoms. Blood samples were collected to determine levels of plasma antibodies.

### Measures

Disease activity was assessed using the Disease Activity Score (DAS)-28 (17) with C-reactive protein (CRP), which includes assessment of the number of swollen and tender joints (out of the 28), CRP, and a visual analog global assessment of health. A DAS-28 of less than 2.6 indicates remission, 2.6–3.2 indicates low disease activity, 3.2–5.1 indicates moderate disease activity, and greater than 5.1 implies severe disease activity.

An arthritis flare is defined as the worsening of the arthritis disease process. The Outcome Measures in Rheumatology has a Rheumatoid Arthritis-Flare Questionnaire (OMERACT-FQ) (18). It was adapted for use with CHIKV arthritis patients. It contains five items to rate pain, physical function, stiffness, fatigue, and participation over the past week using 11-point numeric rating scales (0 = none to 10 = severe) where the composite score is the sum of the responses from the five domains ranging from 0 (no flare) to 50 (extreme flare). In general, flares are indicated by a total score >25, more than a week of symptoms, and patient classification of a “flare,” but analysis as a continuous variable is recommended, which was used in this study.

Since pain is of specific importance to quantifying CHIKV arthritis impact as it is associated with multiple domains of quality of life (19), we measured pain with a separate outcome. Pain intensity was assessed on a visual analog scale from 0–100 by the patient.

The Health Assessment Questionnaire (HAQ) Disability Index (20) was used to measure physical function, composed of a four-level difficulty scale for each item that is scored from 0 to 3, representing normal/no difficulty (0), some difficulty (1), much difficulty (2), and unable to do (3). There are 20 questions in eight categories of functioning – dressing, rising, eating, walking, hygiene, reach, grip, and usual activities. The value of the HAQ index can be interpreted in terms of three categories: mild difficulties to moderate disability (0–1), moderate to severe disability (1–2), and severe to very severe disability (2–3). Disability measured by the HAQ has repeatedly been correlated with mortality rates, progression of aging, and healthcare resource utilization.

### Sample collection and management

Blood was collected by venipuncture into K2EDTA vacutainers. The blood samples were centrifuged at room temperature (18°C–25°C) in a horizontal rotor for 20 minutes at 1,500 relative centrifugal force. Plasma was removed from the blood collection tubes and frozen at −80°C until analyzed.

### Data management

All patients were assigned a unique patient identification number, which was used in the database and for labeling patient samples. All patient data were free of personal identifiers and were stored in the REDCap database at The George Washington University.

### CHIKV serologic confirmation

An immunofluorescence-based assay was used to determine the presence or absence of anti-chikungunya virus IgG antibodies in enrolled patients (EUROIMMUN AG, Germany). Plasma samples were used to coat slides fitted with biochips containing chikungunya positive and negative cells. If present in the sample, the IgG antibody reacted with the positive cells and fluoresced. Slides were read using the 488 nm excitation laser and a 4× objective on a Biotek Lionheart LX fluorescent microscope. This assay has a sensitivity of 97% and specificity of 96%. Patients with negative CHIKV IgG were excluded from the analysis.

### Antibody measurement

The presence of antibodies, including rheumatoid factor (RF) IgM, anti-cyclic citrullinated peptide (CCP), anti-citrullinated α-enolase peptide 1 (CEP-1), anti-citrullinated vimentin (Sa), and immunoglobulins produced in response to chikungunya (IgG and IgM), Zika IgG, and Mayaro IgG, was measured in patient plasma using ELISA (EUROIMMUN AG, Lübeck, Germany) according to the manufacturer’s instructions. Samples were diluted 1:101 in sample buffer and plated either manually or automatically using the Sprinter XL (EUROIMMUN AG, Lübeck, Germany). For determination of RF, samples were diluted automatically 1:201 in sample buffer. Antibodies to CEP-1, Sa, RF, chikungunya, Zika, and Mayaro were measured with a cut-off of 20 relative units (RU)/mL. Antibodies to CCP were measured with a cut-off of 5 relative units (RU)/mL.

### Anti-nuclear antibody (ANA) measurement

Detection of ANA was performed by immunofluorescence assay (IFA) using HEp-20-10 cells from EUROIMMUN AG, Lübeck, Germany (IFA 40: HEp-20-10). HEp-20-10 cells feature an increased number of cells in the mitotic phase and of human nuclear antigens when compared with traditional HEp-2 cells, which aids in pattern interpretation. Samples and slides were processed using the EUROLabWorkstation (EUROIMMUN AG, Lübeck, Germany), followed by automated evaluation using the EUROPattern Software (EUROIMMUN AG, Lübeck, Germany). Serum samples were diluted in PBS-Tween and screened for ANA at 1:40. Positive samples (1:80 and above) were reflexed for titers at three dilutions (1:80, 1:320, and 1:1,280) by automated dilution. The fluorescein isothiocyanate-labeled anti-human IgG conjugate solution contains propidium iodide as counterstain for image segmentation by EUROPattern. Results for ANA patterns were confirmed by an experienced technologist.

Confirmation of ANA and titer determination was performed using the ANA Profile 3 plus DFS70 immunoblot test (EUROIMMUN AG, Lübeck, Germany), which detects the following antigens: nRNP, Sm, SS-A (native), Ro-52, SS-B, Scl-70, PM-Scl, Jo-1, CENP B, PCNA, dsDNA, nucleosomes, histones, ribosomal P-protein, AMA M2, and DFS70. Blot strips were run automatically on the EUROBlotOne (EUROIMMUN AG, Lübeck, Germany) processor.

### Sample size and power

Due to logistical issues related to the COVID-19 pandemic, the final available sample size for the current analyses was *N* = 144. The power for detection of Spearman rank correlations between antibody and arthritis measures was approximated through Pearson correlation (ρ) power calculations. With *N* = 144, there is 80% power to detect ρ = 0.22 (alpha = 0.05, 2-sided). For detecting mean differences between the IgM+ (*n* = 9) and IgM- (*n* = 135), there is 82% power to detect a large, standardized effect size of 1.0 (alpha = 0.05, two-sided).

### Statistical analysis

Frequency distributions were checked visually. Because several measures were skewed, correlations were estimated and tested using the Spearman rank method. Statistical significance was determined as *P* < 0.05 (two-sided, unadjusted) and through Bonferroni adjustment with *P*-value cutoffs determined based on the number of tests within a table. Antibody values below the limit of detection were recoded to 0 or one half of the range between 0 and the lower limit. Means are presented as ±standard deviations. There were no missing data except for one patient with an unknown educational level. Analyses were conducted using SAS version 9.4 with SAS/STAT 15.3 (SAS Institute Inc).

## RESULTS

### Study sample demographics

The study sample was predominantly female with an average age of 48 ± 16 years (Table 1). Educational attainment was included in the demographics as a proxy for socioeconomic status. Most had at least secondary school education. The group had a median of 57 months since CHIKV infection. Ninety percent of the study population reported current joint pain related to their CHIKA, and only 6% reported a history of any sort of arthritis prior to chikungunya infection.

**TABLE 1:** Patient characteristics (*N* = 144)

Characteristic	Value
Age in years mean (SD)	48 (16)
Percent female	80% (115/144)
Percent secondary education or higher	64%
Months since infection median (min, max)	57 (0, 145)
Days of fever during initial chikungunya infection median (min, max)	4 (0, 30)
Percent of study population reporting current joint pain	90% (120/144)
Percent with a history of arthritis prior to chikungunya infection	6% (8/144)

### Arthritis characteristics

We measured arthritis disease severity, flare intensity, pain, and disability. Mean scores were moderate for the Disease Activity Score-28 (3.66 ± 1.23), moderate intensity for the current Flare Score (25.42 ± 12.38), moderate pain (61.47 ± 27.23) on visual analog (scale 0–100), and some disability (Health Assessment Questionnaire 0.77 ± 0.58).

### Correlation of autoantibody levels with arthritis

Plasma autoantibody levels of rheumatoid factor IgM, anti-cyclic citrullinated peptide (CCP), anti- citrullinated α-enolase peptide 1 (CEP-1), anti-nuclear antibody (ANA), and anti-citrullinated vimentin (Sa) were measured. Levels of these factors were correlated with arthritis factors in Table 2. Furthermore, there were no significant correlations between demographic factors, such as age, gender, and educational attainment with autoantibody levels.

**TABLE 2:** Spearman correlations between antibody and arthritis measures^*c*^

Antibody measure	Days of fever during initial chikungunya infection	Pain intensity, patient-rated	Disease severity by the DAS-28	Arthritis flare intensity	Disability by the Health Assessment Questionnaire
RF IgM	0.16	0.21^*b*^	0.19^*b*^	0.01	0.02
Anti-CCP	0.01	0.07	0.06	0.05	0.07
Anti-CEP-1	−0.04	0.08	0.06	0.04	0.19^*a*^
ANA	0.03	−0.10	−0.05	−0.06	0.07
Anti-SA	−0.07	0.10	0.07	0.01	0.05

^*a*^ *P* < 0.05 (unadjusted for multiple testing). None were statistically significant after Bonferroni adjustment.

^*b*^ *P < 0.01 (unadjusted for multiple testing). None were statistically significant after Bonferroni adjustment.

^*c*^ Correlation estimates with Anti-CCP and Anti-CEP1 may not be reliable because the sample contained only one and two positive cases, respectively.

Rheumatoid factor IgM antibody was positive in 21.53% (31/144) of CHIKA cases. There is a weak positive correlation between rheumatoid factor IgM relative units/mL and patient-related pain intensity (r = 0.21, *P* < 0.01) and arthritis disease activity measured by the Disease Activity Score-28 (r = 0.0.19, *P* < 0.01), but these are no longer significant after Bonferroni adjustment. Furthermore, there was no significant correlation between rheumatoid factor levels and the number of days in initial fever with CHIKV infection, arthritis flare intensity, or disability as measured by the Health Assessment Questionnaire.

Anti-CCP antibody was positive in one CHIKA case, accounting for 0.07% of the cohort. This single patient (Patient 170) is unique among the cohort with a positive rheumatoid factor IgM and positive anti-CEP-1 and titers of anti-CCP antibody eight times the upper limit of normal. Patient 170 had moderate disease activity with a Disease Activity Score-28 of 4.3, which is within one standard deviation of the mean from our cohort with eight tender joints, two swollen joints, and C-reactive protein of 1. The presence of anti-CCP was not correlated with the number of days in initial fever with CHIKV infection, pain intensity, arthritis disease severity, arthritis flare intensity, or disability.

Anti-CEP-1 antibody was positive in two CHIKA cases, accounting for 1.39% of the cohort. These cases (Patient 170 and Patient 30) demonstrated notable pain with pain scores of 80 and 100 on a scale from 0 to 100 respectively but with moderate arthritis disease severity, moderate flare intensity, and moderate disability. Among the arthritis factors, anti-CEP-1 showed a weak correlation with disability (r = 0.19, *P* < 0.05) that was no longer significant after Bonferroni correction.

Anti-nuclear antibodies were positive in 26 CHIKA cases, representing 18.06% of the cohort. The most frequent ANA titer was 1:40 in 9.72%, followed by 1:80 in 4.86% and 1:1280 in 2.08%. The ANA staining pattern in ANA positive cases was found to be nucleolar (34.62%), speckled (26.92%), cytoplasmic (19.23%), centromere (7.69%), centriole (3.85%), nuclear membrane (3.85%), and spindle apparatus (3.85%). The presence of ANA was not correlated with worse arthritis factors.

Anti-Sa antibodies were found to be positive in two CHIKA cases, representing 1.39% of the cohort. The presence of anti-Sa antibodies was not correlated with worse arthritis factors.

### Correlation of antiviral antibodies with arthritis factors

Relative units of IgG immunoglobulins produced in response to chikungunya, Zika, and Mayaro as well as qualitative measurement of chikungunya IgM were measured. No significant correlations were found between these levels of IgG antibody and arthritis factors as shown in Table 3. Furthermore, there were no significant correlations between demographic factors, such as age, gender, and educational attainment with antiviral antibody levels.

**TABLE 3:** Spearman correlations between IgG antibody levels and arthritis measures^*a*^

Antibody measure	Days of fever during initial chikungunya infection	Pain intensity, patient-rated	Disease severity by the DAS-28	Arthritis flare intensity	Disability by the Health Assessment Questionnaire
CHIKV	−0.03	−0.13	−0.16	−0.14	−0.05
Zika	0.06	−0.13	−0.00	0.04	0.07
Mayaro	0.08	0.02	−0.11	−0.07	−0.11
Mayaro and/or Zika	0.08	−0.09	0.03	0.08	0.08

^*a*^ No correlations reached statistical significance.

**Fig 1:**
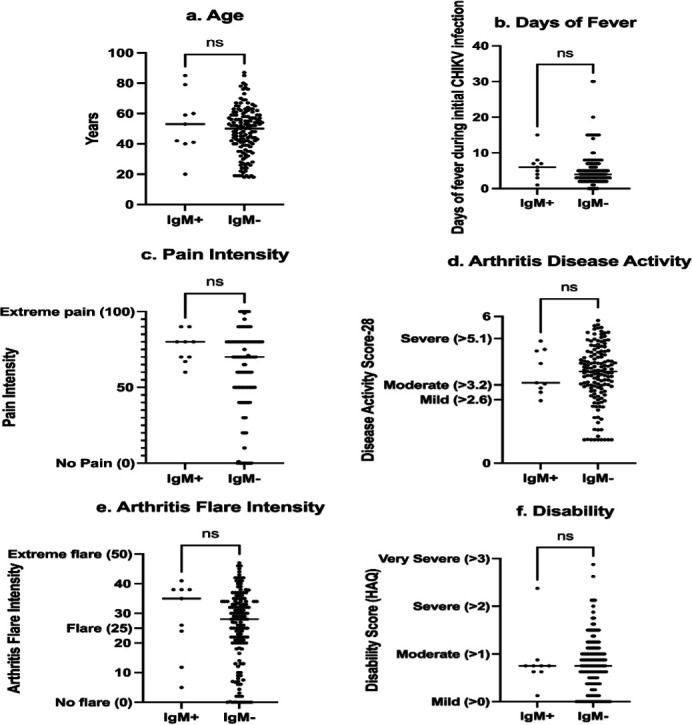
Comparison of demographic and arthritis measures by chikungunya IgM antibody status. Age, days of fever during initial chikungunya infection, pain intensity measured by the visual analog scale from 0 to 100, disease severity as measured by the Disease Activity Score-28, flare intensity measured by the Flare Score adapted for chikungunya (0–50) and disability measured by the Health Assessment Questionnaire were compared between cases positive versus negative for chikungunya IgM. No differences were significant as compared by the Mann–Whitney test (age: *P* = 0.52, days of fever: *P* = 0.35, pain intensity: *P* = 0.10, arthritis disease activity: *P* = 0.89, arthritis flare intensity: *P* = 0.26 and disability: *P* = 0.90).

Chikungunya IgM antibodies were positive in nine CHIKA cases, representing 6.25% of the cohort. The mean duration of time since CHIKV infection in these nine IgM positive cases was 52.7±10.6 months. There was no significant difference in the age, days of initial fever during CHIKV infection, pain intensity, disease severity, flare intensity, or disability between cases with positive IgM versus negative IgM (Fig. 1).

**Fig 2:**
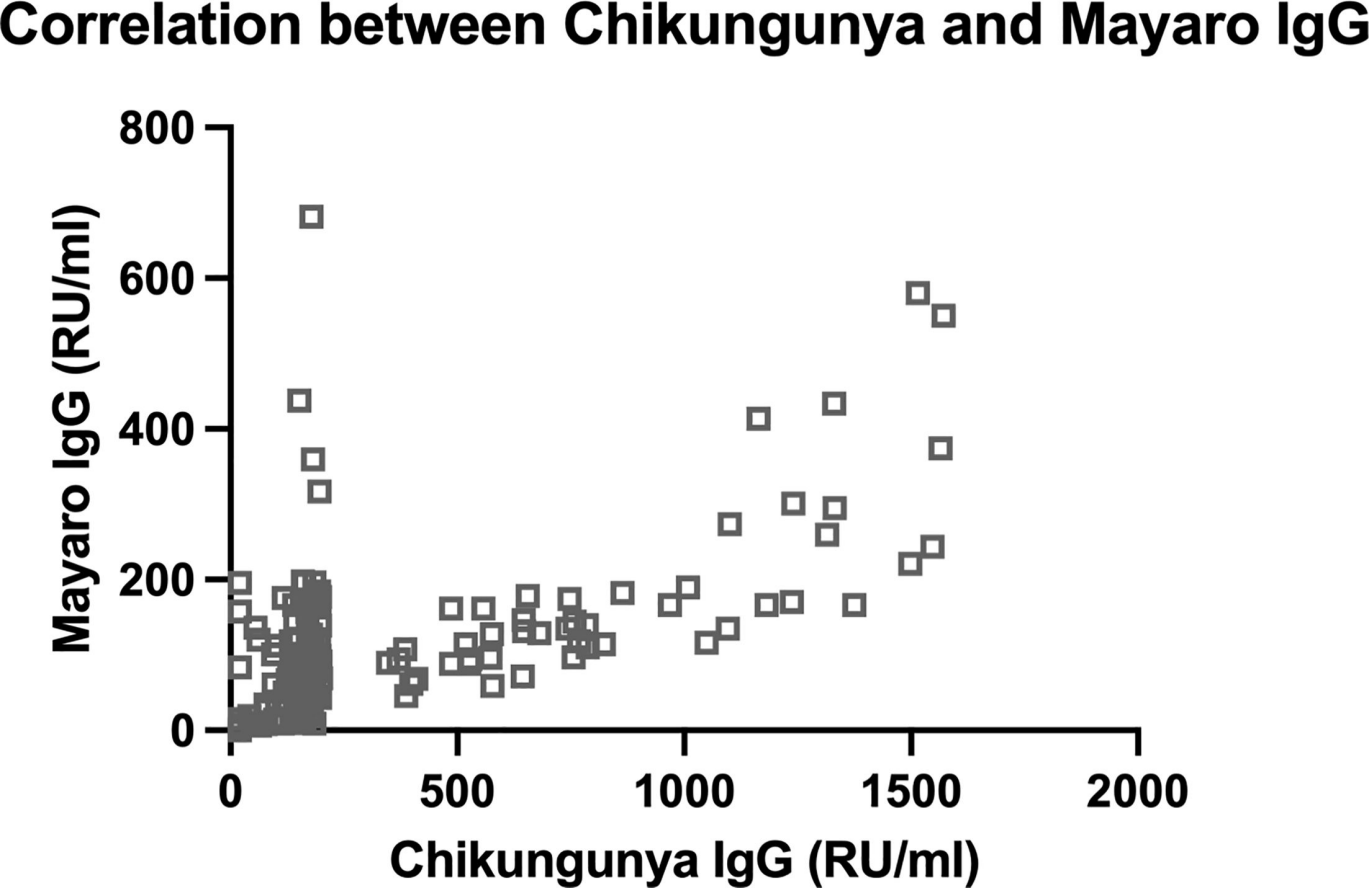
Chikungunya and Mayaro IgG correlation.

Anti-Zika antibodies were positive in 77 CHIKA cases representing 53.47% of the cohort. Anti-Mayaro antibodies were positive in 131 CHIKA cases representing 90.97% of the cohort. Seventy-one cases were positive for both anti-Zika and anti-Mayaro antibodies. The presence of anti-Zika, anti-Mayaro, or both antibodies was not correlated with increased arthritis factors (Table 3). There is a moderate correlation with Spearman r = 0.638 with *P* < 0.0001 between the titer of anti-Mayaro IgG and anti-Chikungunya IgG as shown in Fig 2.

## DISCUSSION

This study systematically assesses the relationship between autoimmune antibodies and chikungunya arthritis disease severity to provide a rigorous evaluation of the potential role of autoantibodies in this chronic post-viral arthritis. The primary finding from this study was that the antibodies we measured were not correlated with arthritis disease severity, flare intensity, pain, or disability, suggesting that levels of these antibodies do not play a critical role in arthritis pathogenesis in CHIKA.

These findings have important implications because they demonstrate that despite similarities, the majority of CHIKA appears to have a unique pathophysiology from seropositive rheumatoid arthritis. In the clinical setting, CHIKA presents similarly to seropositive rheumatoid arthritis, with some CHIKA cases meeting clinical criteria for rheumatoid arthritis (13). However, compared with rheumatoid arthritis where the prevalence of rheumatoid factor is approximately 75% (21), rheumatoid factor and anti-CCP antibodies are present in a substantially smaller percentage of CHIKA cases in multiple cohorts globally, ranging from 1% to approximately 30% (5, 14, 22–26). This study shows for the first time that despite the presence of autoantibodies in our cohort, there is no correlation between levels of these antibodies and measures of clinical arthritis severity. While the prevalence of rheumatoid factor in our cohort was substantially higher (22%) than what is reported in the general population (4%) (27), the levels of the other autoantibodies approximated the levels that are found in the general population with prevalences of anti-CCP of 1% (28), CEP-1 (2%) (29), and ANA (16%) (30) in the general population. Overall, these findings suggest that these autoantibodies are unlikely to provide valuable prognostic information for CHIKA cases, and other measures should be investigated.

These findings are logical in the setting of growing evidence that CHIKA is a T-cell mediated process (31, 32). For example, in CHIKA, large numbers of T cells infiltrate the synovium in CHIKA (33, 34), the presence of T cells is obligatory for the development of arthritis in mouse models (34), and a lack of regulatory T cells is associated with chronic CHIKA (35). Therefore, it appears that CHIKA is primarily driven by T cell-mediated processes as opposed to antibody-related processes; however, the presence of a yet-to-be-discovered autoantibody is possible.

Furthermore, the pathogenesis of chikungunya arthritis involves a complex interplay of immune dysregulation, potential viral persistence, and inflammatory cytokines. Immune dysregulation plays a central role as CHIKV infection triggers an overactive immune response characterized by elevated levels of pro-inflammatory cytokines, such as interleukin-6 (IL-6), tumor necrosis factor-alpha (TNF-α), and C-reactive protein (31). These cytokines contribute to joint inflammation and chronic arthritis in susceptible individuals. While viral clearance typically occurs within weeks, persistent viral RNA or antigens have been detected in synovial tissues, suggesting a potential mechanism for sustained inflammation (33). This persistence may act as a trigger for continued immune activation, even in the absence of active viral replication. Furthermore, histopathological studies of affected joints have revealed infiltration of immune cells, including macrophages and T cells, indicating localized immune dysregulation (33, 34, 36). Collectively, these mechanisms underscore the need for targeted therapies to modulate immune responses, inhibit pro-inflammatory cytokines, and address potential viral persistence in patients with chronic chikungunya arthritis.

RF and ANA were present in approximately one fifth of the cohort, and this is generally higher than the prevalence in healthy persons reported to be up to 4% for rheumatoid factor (27) and 16% for ANA (30). In a cohort of CHIKA cases in Bangladesh, the majority had undifferentiated arthritis, while seven (11.7%) developed rheumatoid arthritis (37). Among the cases with rheumatoid arthritis, two patients were RF positive (14.3%). While reports of positive RF in a small percentage of CHIKA cases are common, Manimunda et al. found no CHIKA cases with positive RF in their cohort (*n* = 203) (38). RF is a non-specific marker of autoimmune disease most commonly found in rheumatoid arthritis and Sjogren’s disease (39). ANA is also a non-specific marker of autoimmune disease most commonly found in lupus and connective tissue diseases (40). The prevalence of ANA in CHIKA cases is not well-established; however, Miner et al. described the presence of ANA in three of nine patients with CHIKV infection returning from Haiti, two of which had high ANA titers (41). RF antibodies and ANAs can also be produced as a result of exposure to an acute or chronic infection, such as hepatitis B or C, HIV, parvovirus B 19, or Epstein–Barr virus (42). Some researchers suggest that infections can act as environmental triggers, inducing or promoting the onset of autoimmune diseases in individuals who are genetically predisposed. In contrast, others believe that the presence of antibodies following infection is a temporary phenomenon and does not ultimately lead to the initiation of autoimmune diseases (42). CHIKV can evade the immune system (43), and CHIKV RNA has been identified to persist in the synovium for months to years after initial infection (33). Therefore, it is possible that the higher ANA and RF levels in our cohort may be the result of chronic exposure to CHIKV. Nevertheless, despite a higher incidence of these autoantibodies compared with the general population, there were no significant correlations with clinical arthritis disease measures after statistical correction for multiple comparisons, suggesting that these antibodies would not be accurate prognostic markers in CHIKA.

In our cohort, the anti-CCP antibody was only elevated in one patient (#170). As anti-CCP is 97% specific for rheumatoid arthritis (44), this suggests that this one patient has rheumatoid arthritis, particularly given that this patient was also positive for rheumatoid factor IgM, anti-CEP-1, and anti-Sa and had titers of CCP eight times the upper limit of normal. Patient 170 had moderate disease activity with a Disease Activity Score-28 of 4.3, which is within one standard deviation of the mean from our cohort. Most likely, this individual has traditional seropositive rheumatoid arthritis given the antibodies; however, there is a hypothesis that citrullinated vimentin in the synovium due to chikungunya replication results in CCP antibodies in CHIKV arthritis and contributes to the development of rheumatoid arthritis (45). Therefore, it is conceivable that the patient developed seropositive rheumatoid arthritis as a result of CHIKV infection, particularly given that the citrullinated vimentin antibody (anti-Sa) was positive in this patient. However, the low prevalence of this antibody in the cohort suggests that this mechanism may not be common in the pathogenesis of most cases of CHIKA.

Anti-CEP-1 was an interesting antibody to examine as it may aid in the early diagnosis of rheumatoid arthritis (46); however, anti-CEP-1 antibodies were also very rare in our cohort, only accounting for 1% of the cohort, therefore limiting the probability that these antibodies play a significant role in arthritis pathogenesis.

As patients living in *Aedes* mosquito endemic areas are often exposed to multiple arboviruses, we also examined if antibodies to chikungunya and other arboviruses were related to arthritis factors. For the past decades, there have been case reports of elevated CHIKV IgM in CHIKA, suggesting that perhaps CHIKV IgM is an indicator of arthritis activity or plays a role in arthritis pathogenesis (14–16). Our findings indicate that CHIKV IgM may not serve as a reliable prognostic or diagnostic marker for arthritis activity, suggesting that IgM antibodies primarily reflect infection rather than direct involvement in arthritis pathogenesis. In a Brazilian cohort, CHIKV-IgM was detectable in 7/57 (12.3%) patients after 28 months of infection and was detected to 35 months post-infection (47). Consistent with our findings, there were no differences in the frequency of chronic musculoskeletal manifestations, and underlying conditions were detected between patients with or without CHIKV-IgM. Similarly, Moro and Borgherini detected CHIKV-IgM in 40% of patients up to 18.7 months after infection, with no association with persistent arthralgia (48, 49). Persistent IgM antibodies have also been observed in other alphavirus infections, such as Ross River virus (RRV) and Sindbis virus (SINV), with detectable levels lasting for years, potentially indicating persistent viral replication or RNA presence (50). For instance, one study reported persistent IgM levels in 19 out of 116 patients (16.4%) lasting between 7 months to 8 years (51)—considerably higher than the 6.25% persistence observed in our CHIKV cohort. This difference may stem from the longer duration post-infection in our analysis. The clinical significance of persistent IgM following RRV and SINV infections remains unclear, and larger cohorts of CHIKV, RRV, and SINV arthritis cases are needed to provide further insights.

Finally, Mayaro virus, another alphavirus known to cause arthritis similar to chikungunya, circulates in the Colombian Amazon region (52) but has not been reported in the Atlántico coastal region where our cohort is located. Our data revealed a significant moderate correlation between Chikungunya IgG titers and Mayaro IgG titers, potentially due to cross-reactivity in the ELISA assay, which has a 76% specificity in another cohort. However, as shown in Fig. 2, not all values are concordant, suggesting either assay variability or the possibility of an undocumented Mayaro outbreak in the Atlántico Department of Colombia. The difficulty in clinically distinguishing between chikungunya and Mayaro virus infections, presence of Mayaro in circulation in the Colombian Amazon region, along with the lack of local diagnostic testing facilities, raises the possibility of a missed outbreak (52). Plaque reduction neutralization testing may be considered in future studies to differentiate Chikungunya versus Mayaro IgG.

### Limitations

There were several limitations in this study. Due to the limited amount of plasma, anti-dengue antibodies were not measured, and chikungunya and Mayaro plaque reduction neutralization tests were not performed. There were insufficient numbers of cases that were anti-Sa positive (*n* = 2), CHIKV IgM positive (*n* = 9), anti-CCP (*n* = 1), and anti-CEP-1 (*n* = 2) to perform a powered analysis of correlation. Therefore, the findings that these do not contribute to arthritis need confirmation with a larger number of positive cases. In addition, given that detection of these antibodies was a rare event, larger sample sizes will also be necessary to detect more subtle potential effects of the antibodies on arthritic disease. Furthermore, there is a lack of a non-infected control group for comparison, and location-specific controls limit the impact. There was no longitudinal analysis of the antibody levels to determine if early levels might predispose to the development of disease. While disease severity is quantified with C-reactive protein and physical examination findings, many of the outcomes are patient-reported without accompanying pathologic or imaging data on the arthritis severity.

In conclusion, this cohort study to date describes the correlation between levels of autoantibodies, viral antibodies, and arthritis outcomes, suggesting that autoantibodies, such as rheumatoid factor, anti-CCP, anti-CEP-1, anti-Sa and ANA, known to play an important role in other autoimmune diseases, do not correlate with chikungunya arthritis relapse disease severity and are unlikely to contribute significantly to arthritis pathogenesis. Furthermore, neither persistent chikungunya IgM nor exposure to other arboviral infections including Zika and Mayaro appeared to be related to worse post-chikungunya arthritis. This suggests that other pathways for arthritis disease pathogenesis should be examined to identify diagnostic and prognostic markers of alphaviral arthritis.

## AFTER EPUB

*[This article was published on 5 March 2025 with an error in an affiliation and in the acknowledgments. The affiliation and acknowledgments were corrected in the current version, posted on 14 March 2025.]*
